# Solution of AntiSeepage for Mengxi River Based on Numerical Simulation of Unsaturated Seepage

**DOI:** 10.1155/2014/270939

**Published:** 2014-02-24

**Authors:** Youjun Ji, Linzhi Zhang, Jiannan Yue

**Affiliations:** ^1^State Key Laboratory of Oil and Gas Reservoir Geology and Exploitation (Southwest Petroleum University), Chengdu 610500, China; ^2^School of Civil Engineering and Architecture, Southwest Petroleum University, Chengdu 610500, China

## Abstract

Lessening the leakage of surface water can reduce the waste of water resources and ground water pollution. To solve the problem that Mengxi River could not store water enduringly, geology investigation, theoretical analysis, experiment research, and numerical simulation analysis were carried out. Firstly, the seepage mathematical model was established based on unsaturated seepage theory; secondly, the experimental equipment for testing hydraulic conductivity of unsaturated soil was developed to obtain the curve of two-phase flow. The numerical simulation of leakage in natural conditions proves the previous inference and leakage mechanism of river. At last, the seepage control capacities of different impervious materials were compared by numerical simulations. According to the engineering actuality, the impervious material was selected. The impervious measure in this paper has been proved to be effectible by hydrogeological research today.

## 1. Introduction 

There are problems of leakage in mass of rivers, lakes, reservoir, and irrigation canals, which bring serious waste of water. The ground water and soil will be polluted when the industrial sewage leaks into soil. Most of the areas of earth surface are located in the arid or semiarid area, so soil in most projects is unsaturated [1–4]. Leakage of river is typical unsaturated seepage. Considering unsaturated seepage in seepage calculation of ground water is much more practical than just thinking of saturated seepage. The boundary of conventional seepage calculation is free water surface and unsaturated seepage is ignored. Mathematic description and parameter determination are complex for unsaturated seepage [5–7].

Study was focused on analytical solution of unsaturated seepage in the 1970s. With the popularization of computer technology, numerical method of unsaturated seepage appeared in the 1980s. From the 1990s, researchers realized that permeability played an important role in improving accuracy of numerical method. The permeability of unsaturated soil had become a hotspot [8, 9].

## 2. Mathematical Model of Ground Water Seepage

In 1856, H. Darcy conducted permeability test of saturated soil and developed Darcy's law 1. Richards thought that Darcy's law was applicative for unsaturated soil but the permeability coefficient was variable, which was the function of moisture volume percentage 2 [10–12]. The equations of motion for unsaturated seepage are

(1) $$\begin{array}{c} v_{i}=K\left(\theta \right)\frac{\partial H}{\partial x_{i}},\;i=x,y,z, \end{array}$$

where *v*_*i*_ is the velocity of the *x*, *y*, and *z* directions, respectively, and *H* is water head.

While ignoring the deformation of unsaturated porous medium, according to principle of mass conservation [13, 14], the equation of continuity is

(2) $$\begin{array}{c} \frac{\partial \left(\rho v_{ii}\right)}{\partial x_{ii}}+\frac{\partial \rho }{\partial t}=0,\;\;i=x,y,z. \end{array}$$

By substituting (1) in (2), we obtain the fundamental equation of unsaturated seepage, which is

(3) $$\begin{array}{c} \frac{\partial \theta }{\partial t}=\frac{\partial }{\partial x_{ii}}\left[K\left(\theta \right)\frac{\partial H}{\partial x_{ii}}\right],\;i=x,y,z, \end{array}$$

where *θ* is moisture volume percentage and *K*(*θ*) is permeability coefficient.

## 3. Numerical Model of Leakage

**Figure 1:**
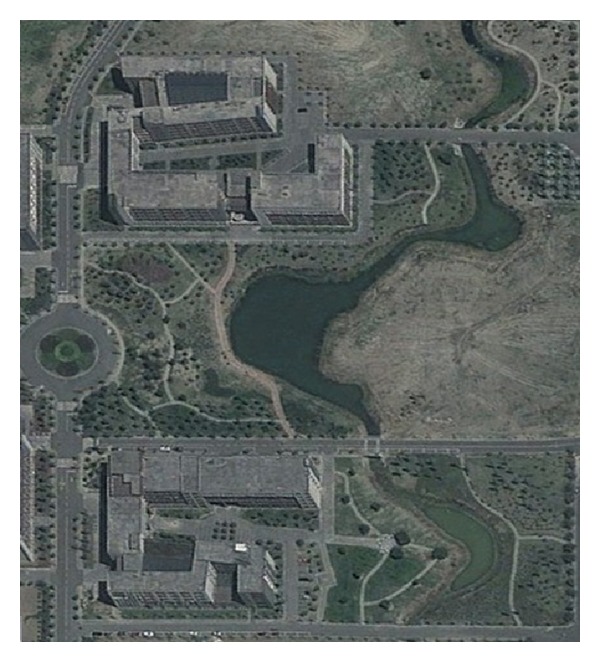
Satellite imagery.

**Figure 2:**
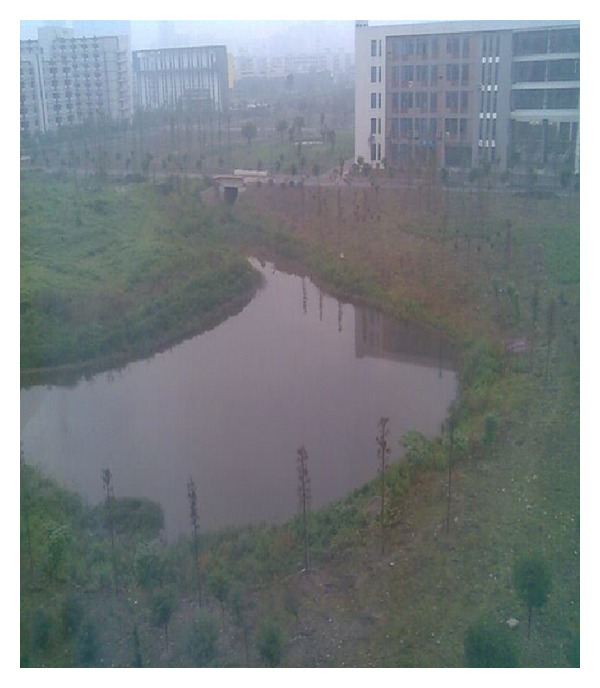
Satellite imagery of study area.

The Mengxi River is located in Chengdu Plain which is in the southwest of China. The Mengxi River is artificial river in the campus of Southwest Petroleum University, whose length is 2 kilometers. The biggest width of river is 15 meters and the smallest width is 2 meters. The water level was 5 meters at first and then leakage phenomenon was serious. The satellite imagery of Mengxi River is shown in Figure 1 and red arrow points to the study area.  Figure 2 is true scene of Mengxi River.

**Figure 3:**
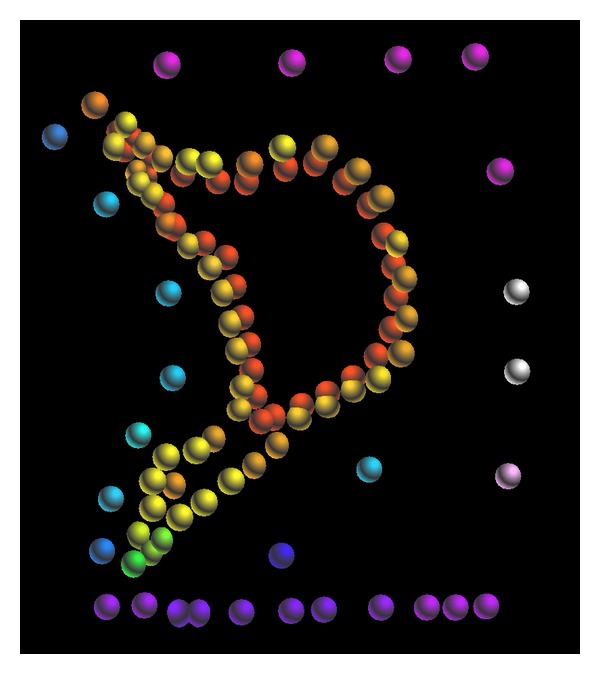
Arrangement plan of measuring points and boreholes.

**Figure 4:**
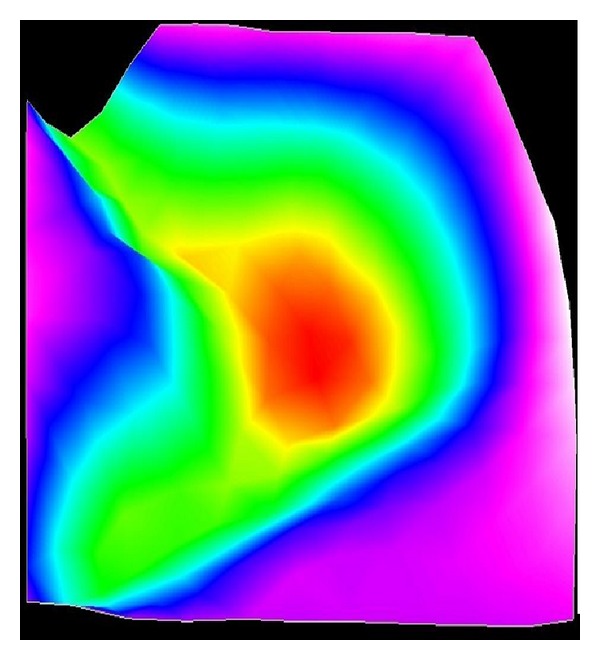
Interpolated topographic map.

In order to measure the topographical map of the Mengxi River, more than 100 points were measured. The place of measuring points and boreholes is shown in Figure 3.  Figure 4 is the interpolated topographic map. There are 18 boreholes, which are evenly distributed among the study area. The study area consists of miscellaneous fill, plain fill, silty clay, sandy silt, fine sand, medium sand, and gravel layer.

**Figure 5:**
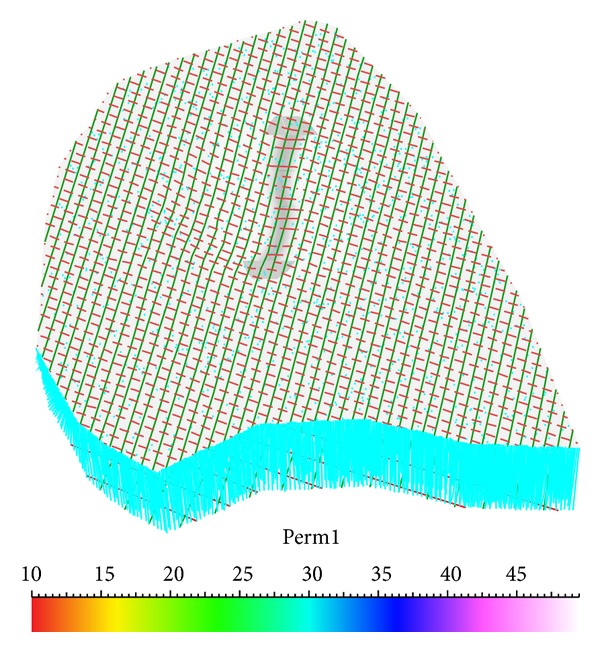
Numerical calculation model.

The three-dimensional geological model was established on the basis of topographic map and geologic examination. The numerical calculation model is shown in Figure 5. The grid number of each direction is 50, 50, and 10.

**Figure 6:**
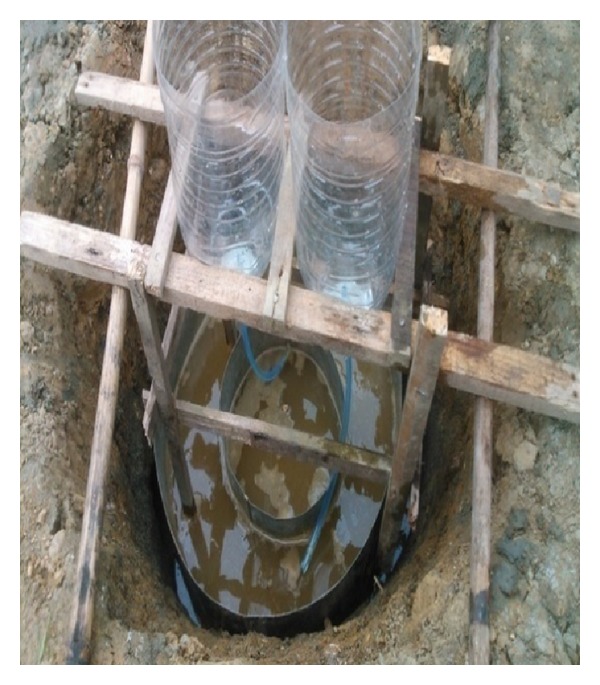
Simple apparatus of double-ring infiltrometer.

The double-ring infiltrometer is common instrument to measure permeability of unsaturated soil on the spot. The double-ring infiltrometer has a higher accuracy than single-ring infiltrometer and test hole. A new simple apparatus of double-ring infiltrometer was developed to test the permeability of soil and 24 pairs of trials were conducted. The simple apparatus is shown in Figure 6. Data in Table 1 is some experimental data of various trials.

The gas-water two-phase flow experiments of unsaturated hydraulic conductivity were carried out by testing instrument of state key laboratory of oil and gas reservoir geology and exploitation, and the characteristic curves of unsaturated seepage were acquired by the experiments. In numerical simulation, instead of the curve of conductivity versus matric suction and the curve volumetric water content of soil versus matric suction, we use the relative permeability curves of water and gas, which simplified the computational process and improved the speed of calculation. On the other hand, the leakage quantity was acquired accurately by integral calculation of seepage velocity. Comparing the calculation result with data of hydrologic exploration, the simulation results are very close to actual situation.

**Figure 7:**
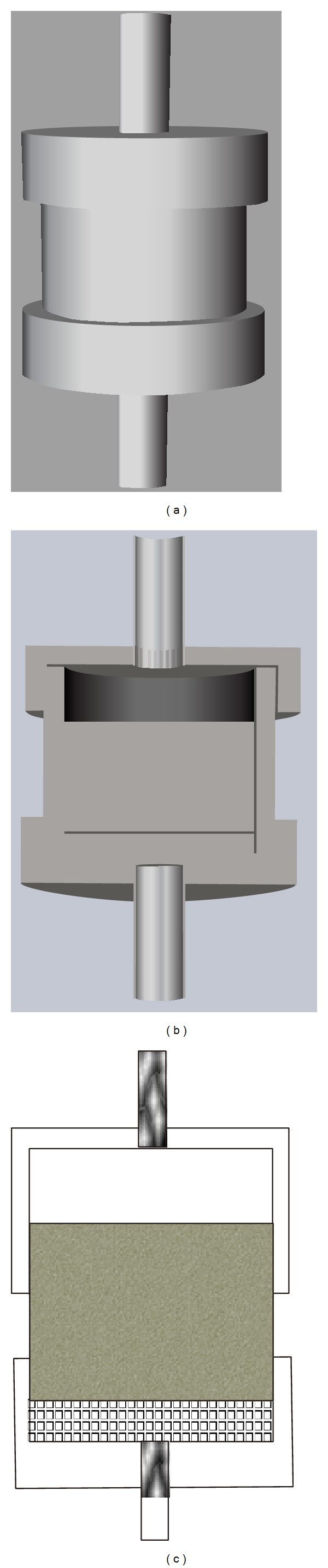
The sketch of equipment.

**Figure 8:**
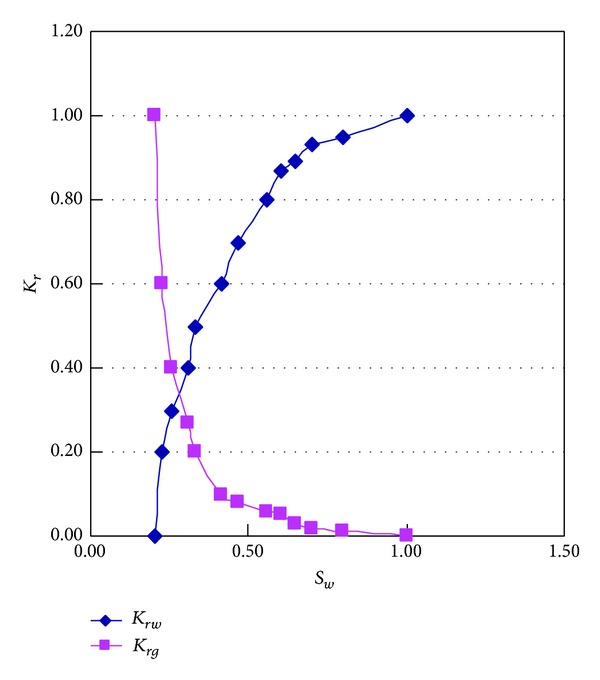
The relative permeability of water versus gas.

As referred to the flow of gas and water in the porous media, the velocity of each phase depends on the their relative volume, which can be described as the relative permeability curve of water and gas, through the equipment (shown in Figure 7) from State Key Laboratory of Oil and Gas Reservoir Geology and Exploitation (Southwest Petroleum University); the results is shown in Figure 8.

## 4. Results and Discussion

The water saturation and pore pressure change with the leakage of water. The initial water saturation of model is 32% and the initial pore pressure is 55 kPa, supposing the water level of the Mengxi River is 3 meter.

**Figure 9:**
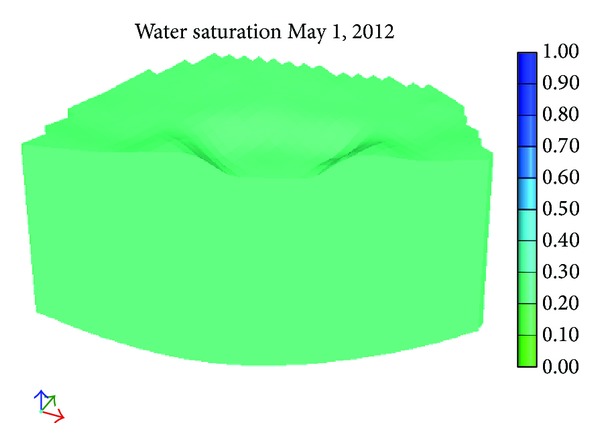
Initial water saturation distribution.

**Figure 10:**
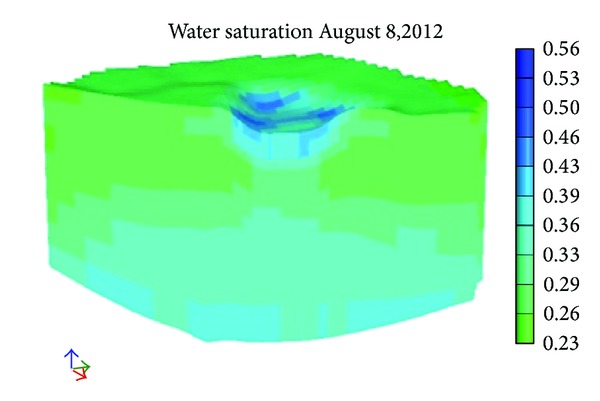
The water saturation after 100 days.

**Figure 11:**
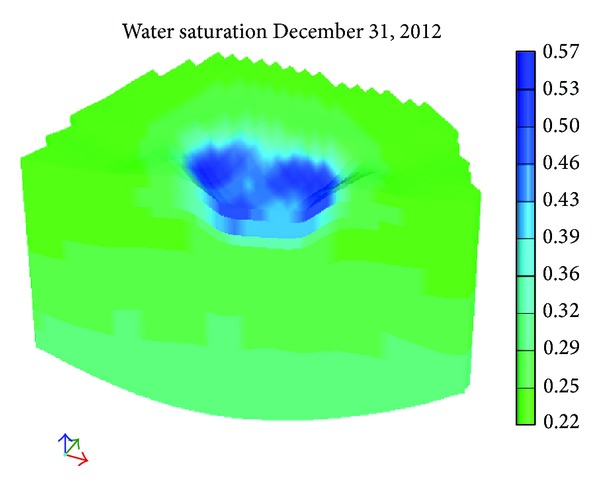
The water saturation after 245 days.

Figure 9 shows the initial distribution of water saturation. Figures 10 and 11 show the distribution of water saturation after 100 days and 245 days, respectively. By Figures 9, 10, and 11, it is obvious that the water saturation increases. The water saturation of area increases from 32% to 65%. The water saturation of area above water level decreases slightly. Reason for this phenomenon is that water in the river and surface soil leaks continuously and the water of lower soil get recharged.

**Figure 12:**
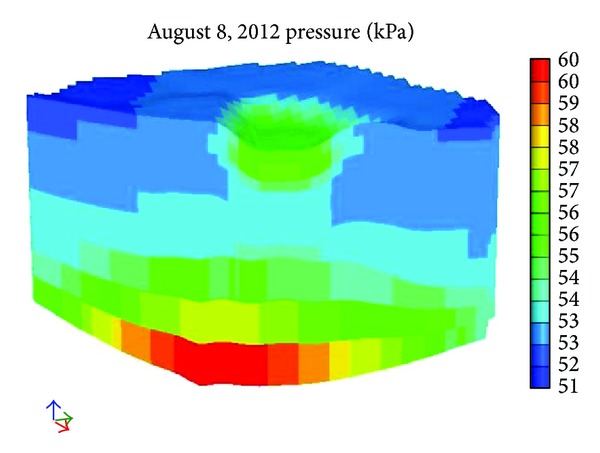
The pore pressure after 100 days.

**Figure 13:**
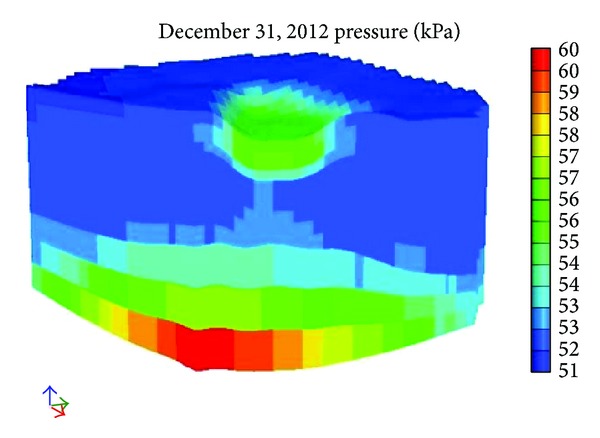
The pore pressure after 245 days.

Figure 12 shows the pore pressure after 100 days and Figure 13 shows the pore pressure after 245 days. With reference to Figures 12 and 13 we can see that pore pressure increases along with depth direction. Another conclusion is that the farther away from the river is, the smaller the pore pressure is. The biggest pore pressure is in the bottom and it runs up to as much as 59 kPa.

**Figure 14:**
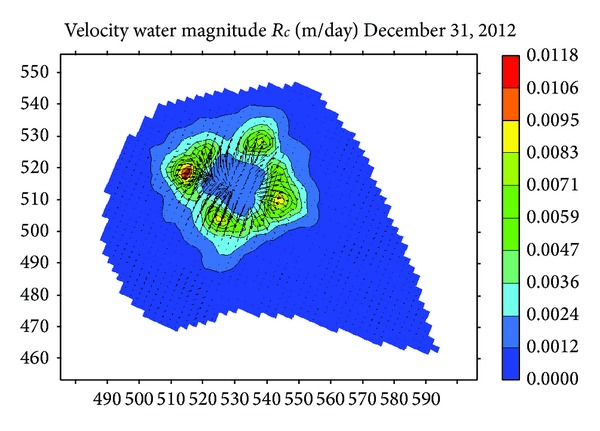
The flow vector of water under river.

Figure 14 shows the velocity vector diagram. The water leaks around from river. The seepage velocity decreases with the increase of distance. Leakage leads to the change of water saturation and pore pressure.

**Figure 15:**
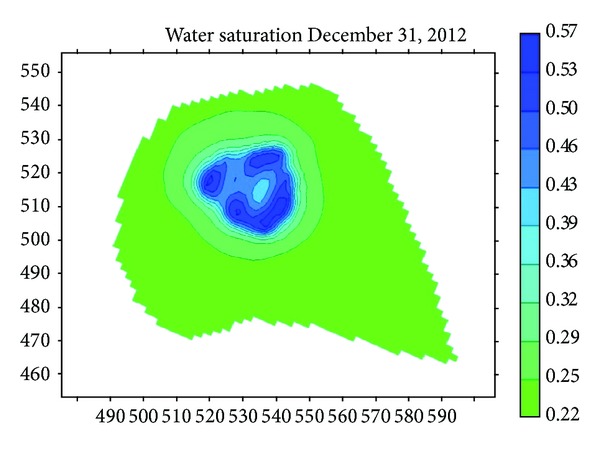
Water saturation with asphalt superstratum.

**Figure 16:**
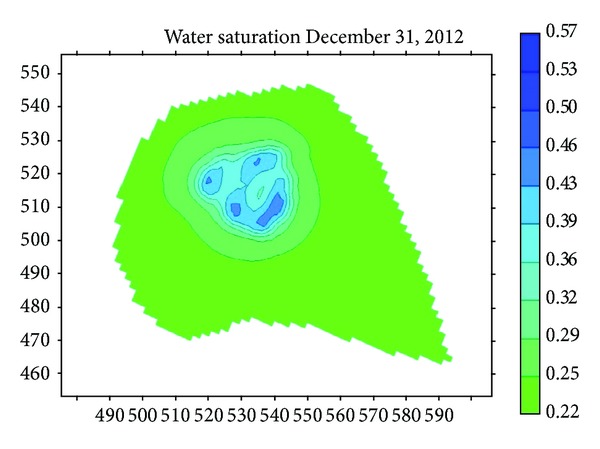
Water saturation with antiseepage film superstratum.

**Figure 17:**
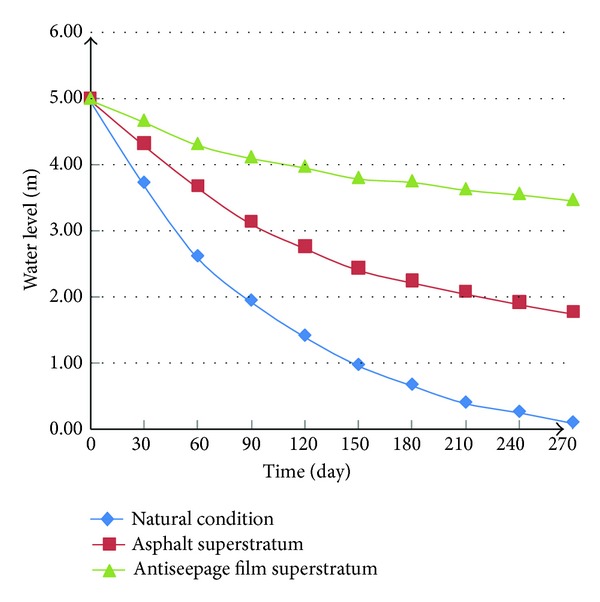
The reduction of water level.

In order to harness the leakage and decrease seepage, the performance of seepage prevention of asphalt and antiseepage film was compared in the study. The biggest water saturation is 51% when the river bed and bank have a superstratum of asphalt shown in Figure 15. The biggest water saturation is 37.4% when the river bed and bank have a superstratum of antiseepage film shown in Figure 15.  Figure 16 shows the curve of monthly drawdown after antiseepage measure.  Figure 17 shows the reduction of water level under different conditions.

## 5. Conclusion

1. The hydraulic conductivity of the soil in Mengxi River was tested by double-ring infiltrometer. The total station was applied to measure the topography of the ground surface surrounding the river. The geological model of Mengxi River was set up by the combination of strata data.
2. Based on seepage mechanics, fluid mechanics, and soil mechanics, the model considering unsaturated seepage of the ground water was established, combined with the hydrological and geological conditions of Mengxi River; the numerical model was built.
3. The leakage of water from Mengxi River was simulated and the results indicate that the leakage was serious. After 245 days, the saturation of the soil under the river increased by 33%, from 32% to 65%; the saturation of soil in the riverbed and slope goes up obviously.
4. The asphalt and impermeable membrane were paved, respectively, to prevent the leakage of water; both of them can reduce the water loss in river, but the effect of the latter is better than the former; when the impermeable membrane was used as waterproof material, the maximal value of soil's saturation under the river only reached 37.4% which is far below the saturation of soil on condition of asphalt as waterproof material.

## Figures and Tables

**Table 1:** Experimental data.

Volume of inject water/mL	Time/min									
	10	20	30	40	50	60	70	80	90	100
Number 1 hole	235	250	180	200	220	250	220	225	200	190
Number 2 hole	180	260	275	270	265	275	270	280	270	270
Number 3 hole	170	260	270	260	245	300	290	150	200	225
Number 4 hole	155	205	215	210	190	185	190	175	180	170
